# Lysozyme Improves the Inhibitory Effects of *Panax notoginseng* Saponins on Phenotype Transformation of Vascular Smooth Muscle Cells by Binding to Ginsenoside Re

**DOI:** 10.3389/fnut.2021.795888

**Published:** 2021-12-23

**Authors:** Yun Huang, Lijian Cui, Hongchao Yang, Ning Chen, Huishan Guo, Xiaoruo Gan, Rong Wang, Weiye Shi, Yu Wu, Yan Zhang, Pin Lv

**Affiliations:** ^1^Beijing Advanced Innovation Center for Food Nutrition and Human Health, Beijing Technology and Business University, Beijing, China; ^2^Cardiovascular Medical Science Center, Department of Cell Biology, Hebei Medical University, Shijiazhuang, China; ^3^Experiment Center, Hebei University of Chinese Medicine, Shijiazhuang, China; ^4^College of Food Science and Biology, Hebei University of Science and Technology, Shijiazhuang, China; ^5^Hebei Food Safety Key Laboratory, Hebei Food Inspection and Research Institute, Shijiazhuang, China

**Keywords:** *Panax notoginseng* saponins, lysozyme, Ginsenoside Re, vascular smooth muscle cells, phenotype transformation

## Abstract

*Panax notoginseng* saponins (PNS) have been used to treat cardiovascular diseases for hundreds of years in China. Lysozyme can bind to exogenous compounds and promote their activity. Nevertheless, knowledge of whether there is a synergistic role between lysozyme and PNS is far from sufficient. In this study, we show that the mixture of PNS and lysozyme synergistically inhibited platelet derived growth factor BB (PDGF-BB)-induced vascular smooth muscle cell (VSMC) viability, and in the five main components of PNS, GS-Re, but not GS-Rb1, NG-R1, GS-Rg1, or GS-Rd, reduced VSMC viability by combined application with lysozyme. Next, the supramolecular complexes formed by GS-Re and lysozyme were detected by mass spectrometry, and the binding ability increased with the concentration ratio of GS-Re to lysozyme from 4:1 to 12:1. In the supramolecular complexes, the relative contents of α-helix of lysozyme were increased, which was beneficial for stabilizing the structure of lysozyme. The 12:1 mixture of GS-Re and lysozyme (12.8 μmol/L GS-Re+1.067 μmol/L lysozyme) repressed PDGF-BB-induced VSMC viability, proliferation, and migration, which were associated with the upregulated differentiated markers and downregulated dedifferentiated markers. Finally, in CaCl_2_-induced rodent abdominal aortic aneurysm (AAA) models, we found that the 12:1 mixture of GS-Re and lysozyme slowed down AAA progression and reversed phenotype transformation of VSMCs. Thus, Gs-Re combined with a small amount of lysozyme may provide a novel therapeutic strategy for vascular remodeling-associated cardiovascular diseases.

## Introduction

Vascular smooth muscle cells (VSMCs), a kind of highly differentiated cells in normal vessels, are quiescent and possess a contractile phenotype. As the main component of media, VSMCs not only provide structural support for vessels, but also play a key role in conferring vascular homeostasis. However, VSMCs are not the terminally differentiated cells, and they can shift from the contractile or differentiated to the synthetic or dedifferentiated phenotype in response to various environmental cues, such as growth factor and mechanical stimulation, manifesting themselves as re-entering the cell cycle and migrating from media into the intima (1, 2), which are the crucial pathological basis of vascular diseases such as atherosclerosis, hypertension, vascular restenosis, arterial aneurysm and diabetic vascular complications (2–4). Therefore, agents that can effectively inhibit VSMC proliferation and migration may have a crucial role in the prevention and treatment of vascular remodeling diseases.

Sanqi (Sanqi Notoginseng Radix et Rhizoma) comprises the dry root and rhizomes of the plant species, *Panax notoginseng* (Burk.) F. H. Chen and has been used to treat various cardiovascular and cerebrovascular diseases for hundreds of years in China (5, 6). *Panax notoginseng* saponins (PNS) is the main active component in Sanqi, and according to the pharmacopeia of China, PNS mainly includes Ginsenoside Rb1 (GS-Rb1) (30%), Ginsenoside Rg1 (GS-Rg1) (25%), Ginsenoside Rd (GS-Rd) (5%), Notoginsenoside R1 (NG-R1) (5%), and Ginsenoside Re (GS-Re) (2.5%). PNS has numerous pharmacological effects, such as blood dynamics invigoration, cerebral vasodilation, hemostasis, anti-inflammation, anti-apoptosis, anti-edema, anti-thromboembolism, anti-coagulation, anti-hyperglycemia, and anti-hyperlipidemia (7, 8).

Lysozyme, known as an alkaline enzyme that can hydrolyze mucopolysaccharides in pathogenic bacteria, has a variety of pharmacological effects such as anti-bacterial, anti-fungal, and anti-inflammation (9–11). Coupled with its biocompatibility, non-irritant and non-toxic to tissues, it has been approved as a food additive and is extensively applied in various food substrates (12). Our study and other studies demonstrated that lysozyme could combine with exogenous compounds through producing allosteric regulation (13–15). One molecule of lysozyme can combine with multiple molecules to synthesize supramolecular complexes with different molecular weights. For example, GS-Rg1 (7.88 × 10^−5^ mol/L) and GS-Re (6.72 × 10^−5^ mol/L) can form 1:1 and 2:1 non-covalent complexes with lysozyme (2.5 × 10^−5^ mol/L) under acidic condition, which may have a certain impact on the natural structure and function of lysozyme (16). The decreased stability of natural proteins is shown as the decreased α-helix structure and the increased surface hydrophobicity. Lysozyme has three α-helix structures, which play key roles in structural stability. Our previous studies found that luteolin and luteoloside influenced the active site of lysozyme in the α-helix (15). Moreover, lysozyme promoted the anti-bacterial activity of baicalin, baicalein, and scutellarin and increased the cytotoxicity of caffeine in HepG2 cells (17, 18). It has been previously reported that PNS inhibited VSMC proliferation by blocking the activation of ERK signaling pathway (19). However, it remains unknown whether lysozyme plays a synergistic role in PNS-repressed VSMC proliferation, and the active ingredients in PNS combined with lysozyme also need to be further investigated.

In the current study, our findings showed that lysozyme promoted the activity of PNS on inhibition of VSMC viability. GS-Re may be the main ingredients of PNS that leads to the decreased VSMC viability through joint application with lysozyme. Next, mass spectral studies show that GS-Re and lysozyme could interact with each other and form supramolecular complexes, which significantly increases the contents of lysozyme α-helix. Finally, the combined use of GS-Re and lysozyme inhibits the proliferation and migration of VSMCs and ameliorates AAA progression.

## Materials and Methods

### VSMC Culture and Treatment

The mouse VSMC line MOVAS was purchased from ATCC, USA (CRL-2797, USA) and cultured in high-glucose Dulbecco's Modified Eagle Medium (DMEM) containing 10% fetal bovine serum and 0.2 mg/ml G-418. VSMCs were pretreated with different concentrations of PNS [Melone Pharmaceutical Co., Ltd., China, dissolved in dimethyl sulfoxide (DMSO)], lysozyme (Melone Pharmaceutical Co., Ltd., dissolved in DMSO), or the mixture of PNS and lysozyme for different time, then stimulated with 10 ng/mL PDGF-BB (R&D) for indicated time periods.

### Cell Viability Assay

VSMCs were seeded onto 96-well plates (5 × 10^3^ cells per well) and pretreated with various concentrations of PNS, lysozyme, or the mixture of PNS and lysozyme for different time before stimulation with or without 10 ng/ml PDGF-BB. Cell viability was measured using cell counting kit-8 (CCK-8 assay; ZOMANBIO, Beijing, China).

### Cell Migration Assay

The migration capability of VSMCs was measured by the wound-healing assay and transwell migration assay.

For the wound-healing assay, directional cell migration of VSMCs was determined in a monolayer using an *in vitro* scratch wound as previously described (20). After achieving confluence, VSMCs were pretreated with GS-Re, lysozyme, or the mixture of GS-Re and lysozyme for 6 h, and then subjected to injury using 200-μl sterilized pipette tips, washed, and stimulated with 10 ng/ml PDGF-BB for 24 h or not. Next, VSMCs were fixed with 4% paraformaldehyde for 30 min and stained by 0.1% crystal violet for 10 min. Three different fields of migration were photographed with a microscope (Leica).

The transwell assay was performed using transwell chambers (8-μm pores, Corning Costar). The VSMCs were pretreated with GS-Re, lysozyme, or the mixture of GS-Re and lysozyme for 6 h, then seeded into the upper chamber, and the lower chamber was filled with DMEM containing 10 ng/ml PDGF-BB or not. Next, VSMCs were allowed to migrate for 10 h, and then fixed with 4% paraformaldehyde for 30 min. After careful removal of the cells on the upper surface with a cotton swab, the cells adhered to the lower surface of the transwell membrane were stained with 0.1% crystal violet for 10 min. Then, five images of each membrane (the center and four quadrants) were captured under an inverted microscope (Leica) for quantification.

### Western Blot Analysis

Cells were prepared with lysis buffer (1% Triton X-100, 150 mM NaCl, 10 mM Tris–HCl, pH 7.4, 1 mM ethylenediaminetetraacetic acid, 1 mM egtazic acid, pH 8.0, 0.2 mM Na_3_VO_4_, 0.2 mM phenylmethylsulfonyl fluoride, and 0.5% Nonidet P-40). Equal amounts of protein (60–100 μg) were separated by 10% sodium dodecyl sulfate–polyacrylamide gel electrophoresis and electro-transferred onto a polyvinylidene fluoride membrane. Membranes were blocked with 5% non-fat milk in tris-buffered saline-Tween for 2 h at room temperature and incubated with specific primary antibodies against proliferating cell nuclear antigen (PCNA; 1:1,000, ab92552; Abcam, Cambridge, United Kingdom), matrix metallopeptidase 9 (MMP-9; 1:1,000, sc-13520, SantaCruz), SM22α (1:1,000, ab14106, Abcam), SM-α-actin (1:1,000, ab7817; Abcam), or α-Tubulin (1:1,000, ab7291; Abcam) at 4°C overnight. The membranes were then incubated with IRDye800® conjugated secondary antibody (1:20,000, Rockland, USA) for 1 h at room temperature, following scanning with the Odyssey Infrared Imaging System. The protein bands of interest were quantified using Quantity One software (Bio-Rad, CA, USA).

### RNA Isolation and Quantitative Real-Time PCR (qRT-PCR)

Total RNA was extracted using TRIzol reagent (Invitrogen, MA, USA) and cDNAs were synthesized by reverse transcriptase SuperScript II (Invitrogen). The qRT-PCR was performed using SYBR Green Real-time PCR Master Mix (Invitrogen) and 7300 Real Time PCR System (Applied Biosystems, Carlsbad, CA, USA). For RT-PCR analysis, the following specific primers were used: PCNA forward, 5′-TTTGAGGCACGCCTGATCC-3′ and reverse, 5′-GGAGACGTGAGACGAGTCCAT-3′; MMP-9 forward, 5′-CTAGTGAGAGACTCTACACGGAG-3′ and reverse, 5′-GAGCCACGACCATACAGATACTG-3′; SM22α forward, 5′- GCCCAGACACCGAAGCTA-3′ and reverse, 5′- CTGCTGCCATATCCTTACCTT-3′; SM-α-actin forward, 5′-CAGGGAGTAATGGTTGGAATGG-3′ and reverse, 5′-GCCGTGTTCTATCGGATACTTCAG-3′; β-actin forward, 5′-TGGAATCCTGTGGCATCCATGAAAC-3′ and reverse, 5′-TAAAACGCAGCTCAGTAACAGTCCG−3′. The samples were pooled to analyze using the qRT-PCR in triplicate, and averaged by experiment, three times repeatedly (*n* = 3).

### Spectral Analysis

AB SCIEX QTRAP3200 mass spectrometer (American Rockwell Allen-Bradley) was used in mass analysis, set in positive ion mode with the conditions of electrospray ionization mass spectrometry soft enough to study the weak non-covalent interactions. The instrumental parameters were set as follows: detector voltage 3 kV, capillary temperature 200°C, the ionization temperature 300°C, sheath gas (nitrogen) flow 35 arb, sweep gas (nitrogen) flow10 arb, and scan range m/z 200–2,000. Each sample was directly injected *via* a syringe pump at the rate of 2 ul/min, and the mass spectrum was cumulated for 5 min. Masses were annotated and processed with PeakView. Four milliliter solution of lysozyme [Melone Pharmaceutical Co., Ltd., dissolved in a 5 × 10^−2^ mol/L Tris–HCl buffer solution (pH 7.40), containing 5 × 10^−2^ mol/L NaCl]/GS-R (Melone Pharmaceutical Co., Ltd., dissolved in 50% methanol) at a molar ratio of 1:4 to 1:12 for mass spectroscopic investigation was incubated for 30 min at 298 K, of which the final concentration was lysozyme 5.0 × 10^−5^ mol/L and saponin from 2 × 10^−4^ to 6 × 10^−4^ mol/L. The data were processed in PeakVew 1.2.

By comparing the first-order mass spectra of lysozyme and non-covalent compound, the number of molecules of compound integrated by lysozyme (*N*) can be calculated to screen out which component was integrated by lysozyme with the highest degree using the following equation:


$$\begin{array}{l} N=n_{\text{charges}}\times \left(m/z_{\text{complex}}-m/z_{\text{lysozyme}}\right)/M \end{array}$$

where *m*/*z*_complex_ and *m*/*z*_lysozyme_ are the ratio of mass to nucleus of non-covalent complex and that of lysozyme, *n*_charges_ the number of charges, and M molecular weight of saponin compound.

Attenuated total reflectance Fourier transform infrared spectroscopy (ATR-FTIR; FTIR-8400s spectrophotometer from Shimadzu Corporation, and horizontal attenuated total reflection accessory from American Perkin Elmer) spectra investigation with the resolution of 4 cm^−1^ and 60 scans were recorded in the range from 1,000 to 2,000 cm^−1^. The spectra of Tris-HCl buffer solution, lysozyme solution (1 × 10^−5^ mol/L), GS-Re solution (1 × 10^−5^ mol/L), and lysozyme solution (1 × 10^−5^ mol/L) with 1 × 10^−5^ mol/L GS-Re were collected after the sample compartment was purged with dry air to eliminate the absorption of water vapor. The differential spectrum of pure lysozyme was obtained from the absorbance spectrum of lysozyme solution subtracted by that of Tris–HCl buffer solution, and the spectrum of GS-Re solution was subtracted from that of GS-Re-lysozyme to obtain the lysozyme (after GS-Re was added) differential spectrum. The data were analyzed and graphed in Origin 9.0. The subtractive spectra were subjected to baseline correction within the scope of 1,700–1,600 cm^−1^ amide I band and smoothed with Savitzky-Golay, then fitted by the Gauss curve fitting with the second derivative and Fourier deconvolution was applied to estimate the position of peaks and half-peak width. The secondary structure contents of lysozyme were calculated according to the absorption bands observed in the amideIregion corresponding to α-helix (1,642–1,660 cm^−1^), β-sheet (1,613–1,637 cm^−1^ and 1,682–1,689 cm^−1^), β-turn (1,662–1,700 cm^−1^), and disordered (1,637–1,645 cm^−1^) structures (21).

### Mouse Model of AAA

Male C57BL/6J mice, aged 8–12 weeks, were subjected to surgery in accordance with the CaCl_2_-daubed abdominal aortic aneurysm (AAA) model as previously described (22–24). In brief, the mice were anesthetized and the infrarenal abdominal aortas were surrounded with a small piece of gauze soaked in CaCl_2_ (0.5 mol/L) for 15 min, followed by another piece of gauze soaked in saline for 5 min. Mice receiving a single treatment of saline for 15 min were used as a control group. The mice were sacrificed 21 days later, and the abdominal aorta between the renal arteries and bifurcation of the iliac arteries was isolated from the surrounding retroperitoneal structures. The diameter of the aorta was measured with video microscopy in triplicate midway between the renal artery origin and iliac artery bifurcation. GS-Re (50 mg/kg/day) or the mixture of GS-Re and lysozyme (50 mg/kg/day, the molar ratio of GS-Re:lysozyme was 12:1) was orally administered by gastric gavage from 7 days before CaCl_2_ treatment to 21 days after the CaCl_2_ treatment (*n* = 8 per group). All animal procedures conformed to the Guide for the Care and Use of Laboratory Animals published by the National Institutes of Health and were approved by the Institutional Animal Care and Use Committee of Hebei Medical University.

### Hematoxylin and Eosin (H and E) Staining and Immunohistochemistry

The aortas were fixed with 4% polyoxymethylene, dehydrated by ethanol, and placed in xylene, and then embedded in paraffin wax and sliced. The sections were baked at 65°C for 4 h, dewaxed, hematoxylin stained for 15 min, and decolorized with hydrochloric acid alcohol solution for 5 s. Then, the sections were stained with eosin for 2 min and dehydrated. Finally, the sections were mounted in neutral gel and the tissue sections were observed under a microscope.

The immunohistochemical analyses were processed according to standard procedures. In short, the slices were baked and then dewaxed in xylene and hydrated in gradient alcohol. Then, 3% H_2_O_2_ was added to the sections to remove endogenous peroxidase. Immunostained sections were preincubated with 5% normal goat serum and then incubated with specific primary antibodies against PCNA (1:500, ab92552; Abcam), MMP-9 (1:500, sc-13520; SantaCruz, USA), SM22α (1:500, ab14106; Abcam), and SM-α-actin (1:500, ab7817; Abcam). The sections were incubated with the horseradish peroxidase streptavidin biotinylated secondary antibody followed by diaminobenzidine (DAB kit, ZSGB-BIO, China). For the negative controls, the primary antibody was replaced with non-immune rabbit or mouse serum. Staining intensities were determined by measurement of the integrated optical density with light microscopy using a computer-based Image-Pro Morphometric System. The results are expressed as the mean value of at least three randomly chosen slides in each group.

### Statistical Analysis

Data analysis was performed using SPSS version 16.0 or Graphpad Prism 6 software. Data are presented as means ± standard deviation from at least three independent experiments, and each independent experiment was repeated at least three times to obtain the mean. Normally distributed datasets were analyzed with the unpaired Student's *t*-test for two independent groups or paired *t*-test for two dependent groups, and the one-way analysis of variance followed by the post Bonferroni's multiple comparisons test for ≥3 groups. For all statistical comparisons, a value of *P* < 0.05 was considered statistically significant and denoted with one, two, and three asterisks when lower than 0.05, 0.01, and 0.001, respectively.

## Results

### Lysozyme Promoted the Effects of PNS on Inhibition of VSMC Viability

**Figure 1:**
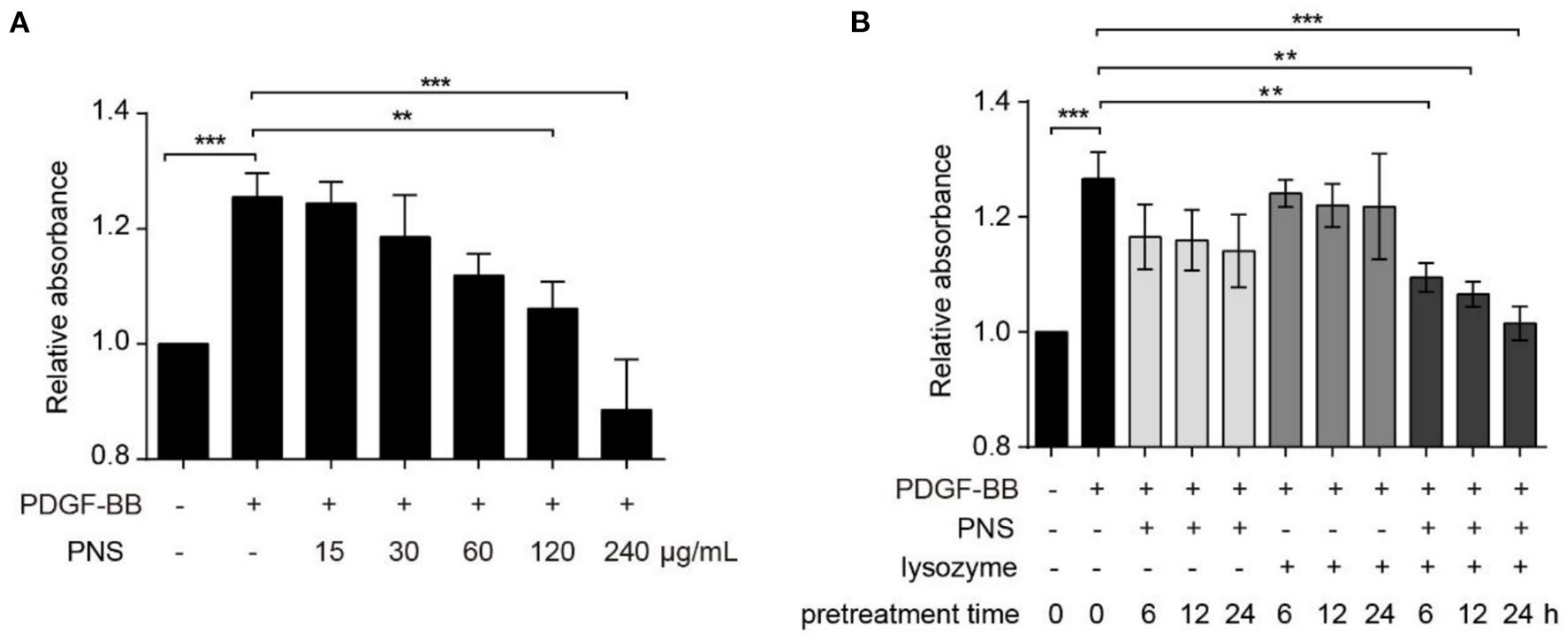
Lysozyme promoted the activity of *Panax notoginseng* saponins (PNS) on inhibition of vascular smooth muscle cell (VSMC) viability. **(A)** Cell counting kit-8 (CCK-8) for the viability of VSMCs, cultured in serum-starved medium containing 15, 30, 60, 120, and 240 μg/mL PNS or dimethyl sulfoxide (DMSO) for 24 h, and then stimulated by platelet derived growth factor BB (PDGF-BB) for 24 h or remained untreated. **(B)** CCK-8 for the viability of VSMCs, cultured in serum-starved medium containing 60 μg/ml PNS, 60 μg/ml lysozyme, or the mixture of PNS and lysozyme (30 μg/ml PNS+30 μg/ml lysozyme) for 6, 12, and 24 h, and then stimulated by PDGF-BB for 24 h or remained untreated. Data are presented as mean ± SD, **P < 0.01, ***P < 0.001.

To investigate the effect of PNS combined with lysozyme on viability of VSMCs, we conducted CCK-8 assays. As displayed in Figure 1A, PDGF-BB significantly promoted VSMC viability, and after pretreatment of VSMC with different concentrations of PNS, 15, 30, and 60 μg/ml PNS for 24 h had no effect on VSMC viability, whereas 120 and 240 μg/ml PNS significantly inhibited cell viability, suggesting that PNS inhibited VSMC viability in a concentration-dependent manner, similar with the previous studies (19).

Next, VSMCs were pretreated with 60 μg/ml PNS, 60 μg/ml lysozyme, and the mixture of PNS and lysozyme (30 μg/ml PNS + 30 μg/ml lysozyme) for 6, 12, and 24 h, respectively. As shown in Figure 1B, PNS or lysozyme used alone had no effect on the viability of VSMC stimulated by PDGF-BB, but the combination of PNS and lysozyme significantly inhibited cell viability in a time-dependent manner. These data indicated that the joint application of PNS and lysozyme repressed viability of VSMCs.

### GS-Re May Be the Main Ingredients of PNS That Contributes to the Decreased VSMC Viability Through Joint Application With Lysozyme

**Figure 2:**
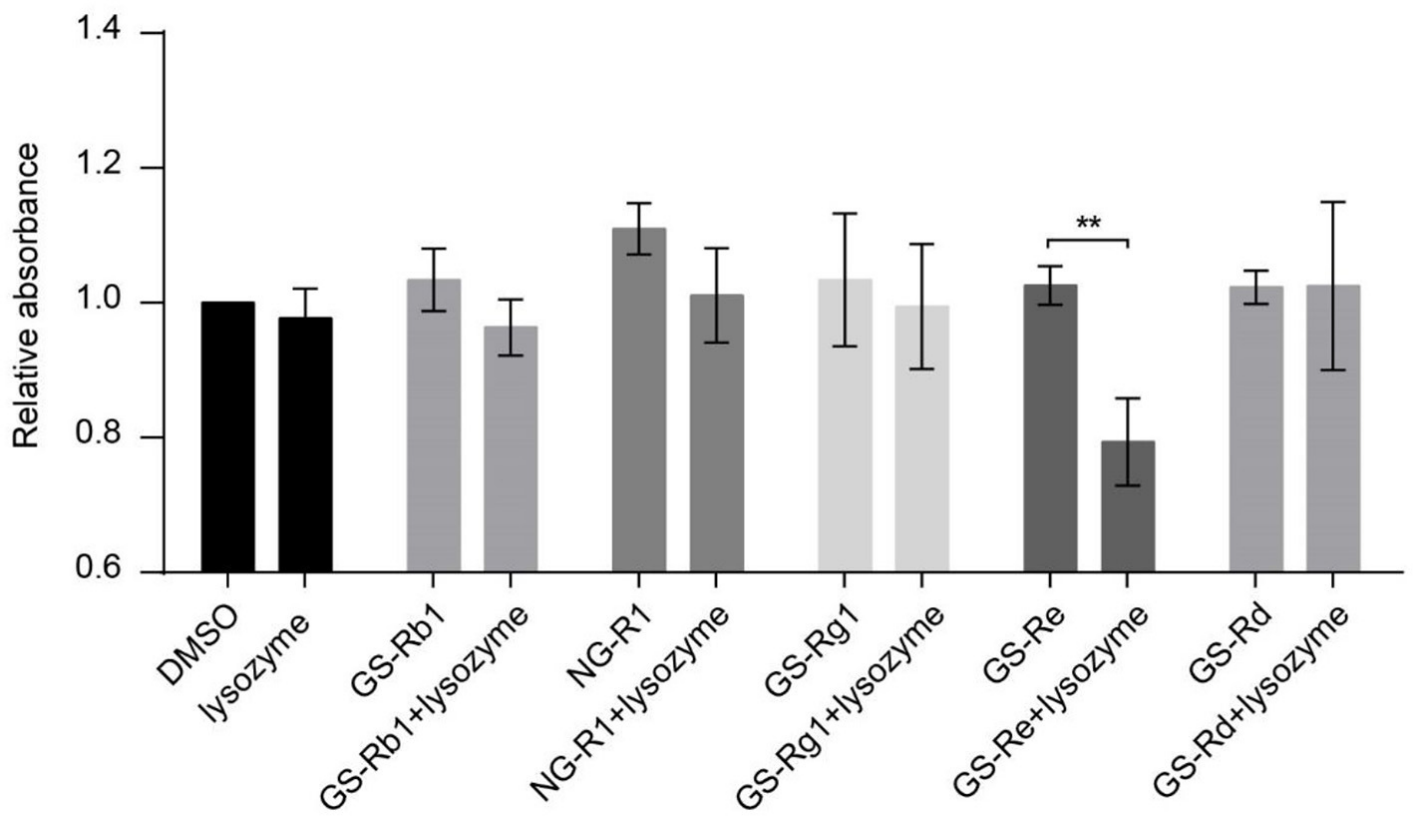
GS-Re may be the main ingredients of PNS that contributes to the decreased VSMC viability through combining with lysozyme. CCK-8 for the viability of VSMCs, cultured in serum-starved medium containing 30 μg/ml lysozyme, GS-Rb1, NG-R1, GS-Rg1, GS-Re or GS-Rd, or the mixture of lysozyme and five ingredients of PNS (15 μg/ml lysozyme+15 μg/ml GS-Rb1, NG-R1, GS-Rg1, GS-Re, or GS-Rd, respectively) for 24 h, and then stimulated by PDGF-BB for 24 h. Data are presented as mean ± SD, **P < 0.01.

To screen the main ingredients of PNS that inhibit VSMC viability through joint application with lysozyme, we determined the effect of the synergism of lysozyme and GS-Rb1, NG-R1, GS-Rg1, GS-Re, or GS-Rd, the five main ingredients of PNS, on VSMC viability. Compared with the DMSO control group, the viability of VSMCs was not changed with the addition of 30 μg/ml lysozyme, GS-Rb1, NG-R1, GS-Rg1, GS-Re, or GS-Rd, and the viability was also not affected by the joint application of 15 μg/mL lysozyme and 15 μg/ml GS-Rb1, NG-R1, GS-Rg1, or GS-Rd. However, this response was significantly attenuated by the synergism of GS-Re and lysozyme (15 μg/mL GS-Re+15 μg/ml lysozyme) (Figure 2), meaning that GS-Re, but not GS-Rb1, NG-R1, GS-Rg1, or GS-Rd, may be the main ingredients of PNS in the joint application with lysozyme.

### Mass Spectral Studies on the Interaction Between GS-Re and Lysozyme

**Figure 3:**
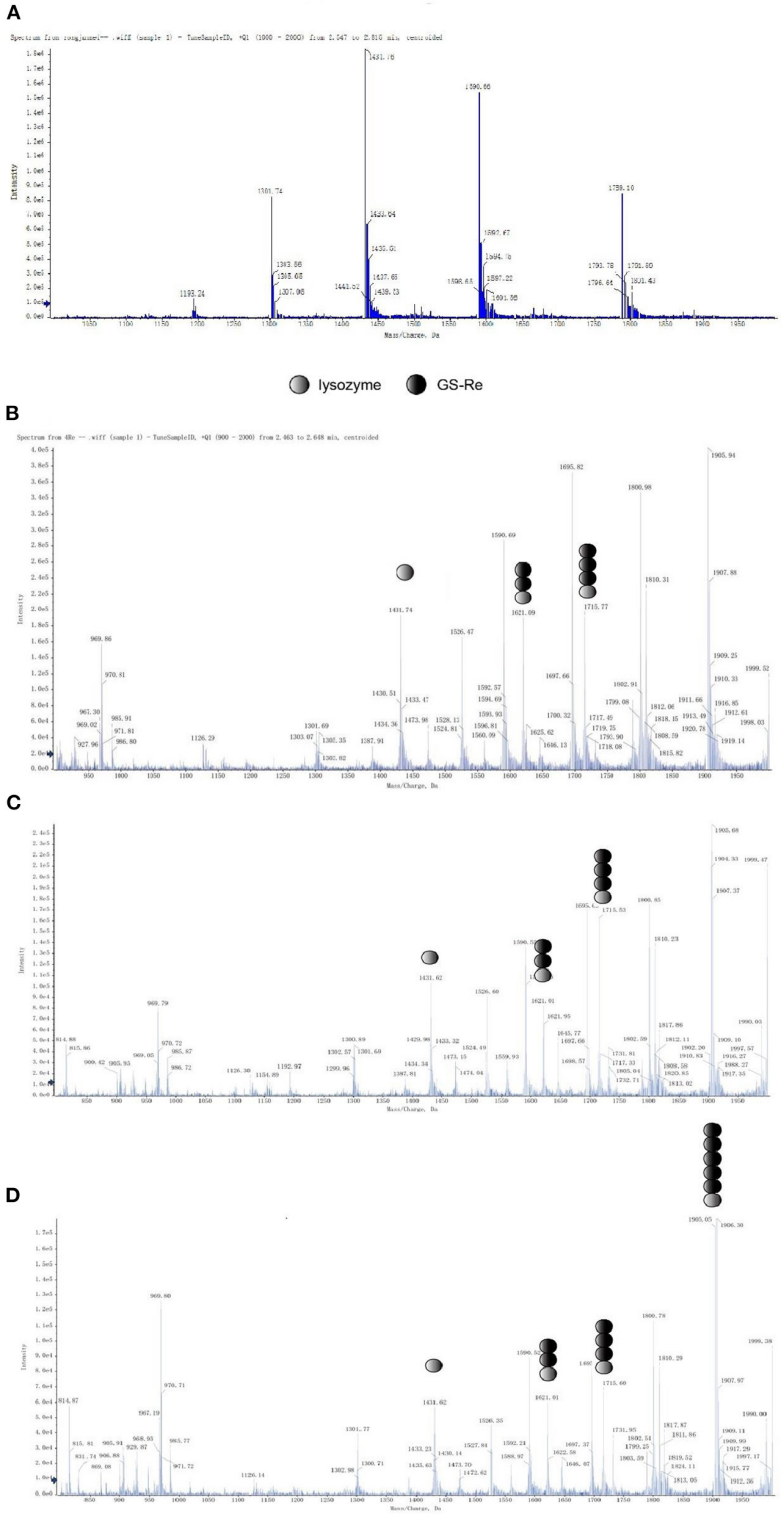
Mass spectral studies on the interaction between GS-Re and lysozyme. The interaction between GS-Re and lysozyme was determined in the condition of detector voltage 3 KV, capillary temperature 200°C, the ionization temperature 300°C, sheath gas (nitrogen) flow 35 arb, and sweep gas (nitrogen) flow 10 arb. **(A)** Lysozyme, *C*_(lysozyme)_ = 5.0 × 10^−5^ mol/L; **(B)** lysozyme+GS-Re, *C*_(lysozyme)_ = 5.0 × 10^−5^ mol/L, *C*_(GS−Re)_ = 2.0 × 10^−4^ mol/L; **(C)** lysozyme+GS-Re, *C*_(lysozyme)_ = 5.0 × 10^−5^ mol/L, *C*_(GS−Re)_ = 4.0 × 10^−4^ mol/L; **(D)** lysozyme+GS-Re, *C*_(lysozyme)_ = 5.0 × 10^−5^ mol/L, *C*_(GS−Re)_ = 6.0 × 10^−4^ mol/L.

A series of mass peaks of different mass to charge ratios, including the peaks at m/z 1,193.2, 1,301.7, 1,431.7, 1,590.6, and 1,789.1, displaying multicharge distribution of lysozyme from (M+8H)8+ to (M+12H)12+, represents the +12, +11, +10, +9, and +8 charge states of lysozyme, respectively, after the lysozyme solution detected by electrospray ionization-mass spectrometry, in which peaks of +10 charge number were the most obvious (Figure 3A). The average molecular weight of lysozyme was 14,306.8, which was close to the theoretical molecular weight.

The molecular ion peaks of various complexes composed of GS-Re and lysozyme were detected by mass spectrometry, suggesting that GS-Re might be combined with lysozyme and the supramolecular complexes were formed (Figures 3B–D). The peaks at m/z 1,621.0 and 1,715 indicated the +10 charge states of 1:2 and 1:3 complex of GS-Re with lysozyme, respectively (Figures 3B,C). In Figures 3C,D, the concentration ratio of lysozyme to GS-Re increased from 1:8 to 1:12, and with the increased concentration ratio, the molecular ion peak intensity ratio of supramolecular complex formed by one molecule of lysozyme and two molecules of GS-Re to free lysozyme increased from 60.7 to 107.7%. Compared with Figures 3B,C, another strong signal at the peak of 1905.5 was detected in Figure 3D, which may be associated with the supramolecular complex with +10 charges formed by one molecule of lysozyme and five molecules of GS-Re. These data suggested the combined ability between GS-Re and lysozyme could be increased with the high concentration ratio of GS-Re to lysozyme.

### Effect of GS-Re on the Secondary Structure of Lysozyme

**Figure 4:**
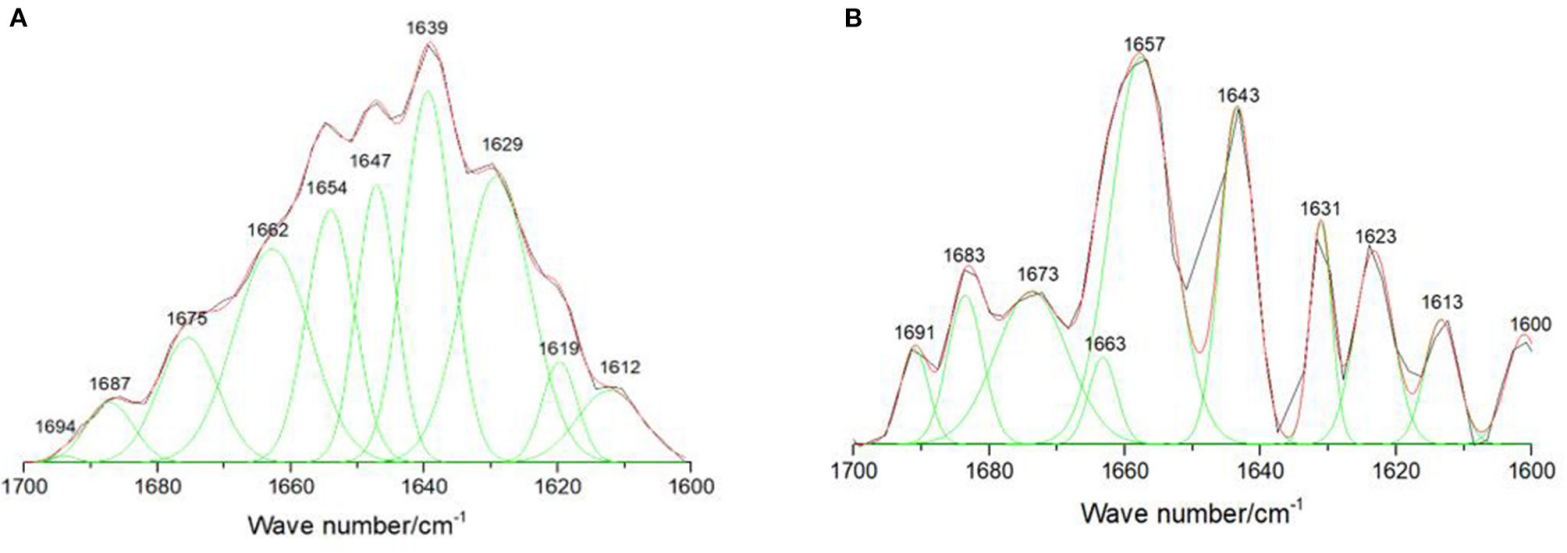
Effect of GS-Re on the secondary structure of lysozyme. The curve-fitting figures of attenuated total reflectance Fourier transform infrared spectroscopy (ATR-FTIR) differential spectra within the scope of 1,700–1,600 cm^−1^ were fitted by the Gauss curve fitting, and the secondary structure contents of lysozyme were calculated from the areas of the individual assigned bands and their fraction of the total area. **(A)** Lysozyme, *C*_(lysozyme)_ = 5.0 × 10^−5^ mol/L; **(B)** (lysozyme+GS-Re)−GS-Re, *C*_(lysozyme)_ = 5.0 × 10^−5^ mol/L, *C*_(GS−Re)_=5.0 × 10^−5^ mol/L. ATR-FTIR curve-fitting differential spectra with the resolution of 4 cm^−1^ and 60 scans in the range from 1,000 to 2,000 cm^−1^.

It is important to study the change of lysozyme α-helix structure, because the active site of lysozyme is mainly in helices region. To further explore whether the formation of this supramolecular complex would influence the secondary structure of lysozyme or not, we performed infrared spectroscopy on lysozyme. The absorption bands in the amide I region corresponding to α-helix (1,642–1,660 cm^−1^), β-sheet (1,613–1,637 cm^−1^ and 1,682–1,689 cm^−1^), β-turn (1,662–1,700 cm^−1^), and disordered (1,637–1,645 cm^−1^) structures were observed in the ATR-FTIR spectra (15, 21). The correlative differential spectra and Gaussian fitting plots of lysozyme and (GS-Re-lysozyme)-GS-Re were shown in Figure 4. The relative contents of lysozyme secondary structure were changed after adding GS-Re. The average relative contents of lysozyme α-helix in the presence of GS-Re significantly increased from 24.1 to 32.4% with β-sheet decreasing from 32.4 to 26.0%, and the significant changes on the lysozyme α-helix and β-sheet relative contents in the presence of GS-Re with *p*-values < 0.05 indicated the interaction between lysozyme and GS-Re induced folding of lysozyme peptides and changes of lysozyme conformation, which may affect the biological function of lysozyme.

### Combined Use of GS-Re and Lysozyme Inhibits the Phenotype Transformation of VSMCs

**Figure 5:**
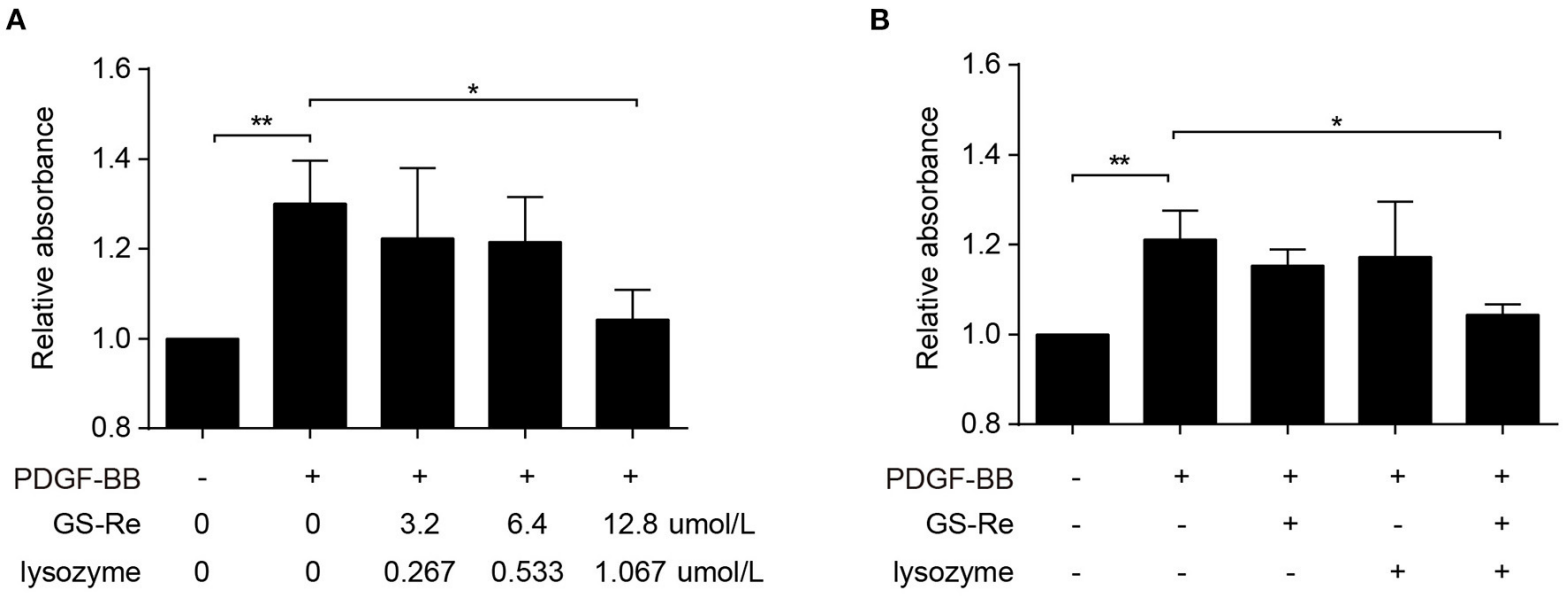
The combined use of GS-Re and lysozyme inhibits the viability of VSMCs. **(A)** CCK-8 for the viability of VSMCs, cultured in serum-starved medium containing the mixture of GS-Re and lysozyme (the molar ratio of GS-Re/lysozyme was 12:1) for 6 h, and then stimulated by PDGF-BB for 24 h or remained untreated. **(B)** CCK-8 for the viability of VSMCs, cultured in serum-starved medium containing 12.8 μmol/L GS-Re,1.067 μmol/L lysozyme or the mixture of GS-Re and lysozyme (12.8 μmol/L GS-Re+1.067 μmol/L lysozyme) for 24 h, and then stimulated by PDGF-BB for 24 h or remained untreated. Data are presented as mean ± SD, *P < 0.05, **P < 0.01.

To further evaluate the effects of GS-Re combined with lysozyme on viability of VSMCs, the mixture of GS-Re and lysozyme (the molar ratio of GS-Re:lysozyme was 12:1; 3.2 μmol/L GS-Re+0.267 μmol/L lysozyme, 6.4 μmol/L GS-Re+0.533 μmol/L lysozyme, and 12.8 μmol/L GS-Re+1.067 μmol/L lysozyme) was used. Results from CCK-8 assay showed that the mixture of 3.2 μmol/L GS-Re+0.267 μmol/L lysozyme and the mixture of 6.4 μmol/L GS-Re+0.533 μmol/L lysozyme had no effect on the viability of VSMCs, and the mixture of 12.8 μmol/L GS-Re+1.067 μmol/L lysozyme significantly decreased the cell viability (Figure 5A), whereas 12.8 μmol/L GS-Re or 1.067 μmol/L lysozyme had no effect on the activity of VSMC (Figure 5B).

**Figure 6:**
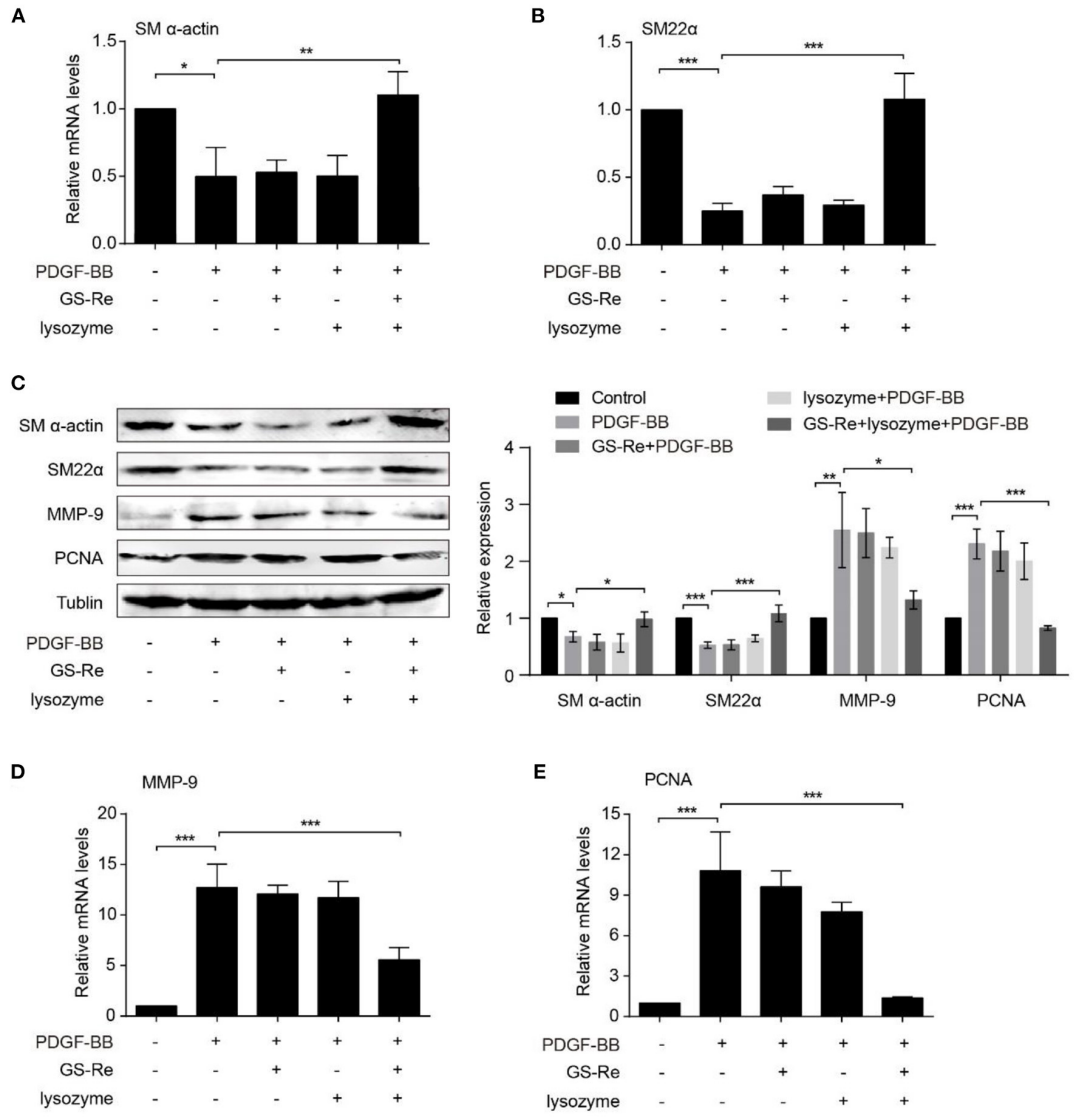
The combined use of GS-Re and lysozyme inhibits the phenotype transformation of VSMCs. Real-time polymerase chain reaction (PCR) **(A, B, D, E)** or Western blot **(C)** analysis of the expression of SM-α-actin, SM22α, matrix metallopeptidase 9 (MMP-9), and proliferating cell nuclear antigen (PCNA) in VSMCs, cultured in serum-starved medium containing 12.8 μmol/L GS-Re, 1.067 μmol/L lysozyme, or the mixture of GS-Re and lysozyme (12.8 μmol/L GS-Re+1.067 μmol/L lysozyme) for 6 h, and then stimulated by PDGF-BB for 6 h (real-time PCR), 24 h (Western blot), or remained untreated. Data are presented as mean ± SD, *P < 0.05, **P < 0.01, ***P < 0.001.

Aberrant proliferation is a hallmark of VSMC phenotype transformation during vascular injury (1, 2). To further investigate the possible mechanisms underlying the effects of PNS and lysozyme synergism on modulation of VSMC phenotype, the expression of SM-α-actin and SM22α, the typical markers of differentiation phenotypes, and the expression of MMP-9 and PCNA, the typical markers of synthetic phenotypes, were examined through qRT-PCR and Western blot. As shown in Figures 6A–C, the expression of SM-α-actin and SM22α were clearly determined in the control group and were significantly downregulated in PDGF-BB-stimulated VSMCs, agreeing with previous studies (20). Compared with PDGF-BB group, the expression of SM-α-actin and SM22α had almost no changes stimulated by 12.8 μmol/L GS-Re or 1.067 μmol/L lysozyme along but increased by 12:1 mixture of GS-Re and lysozyme (12.8 μmol/L GS-Re + 1.067 μmol/L lysozyme). At the same time, PDGF-BB increased the expression of MMP-9 and PCNA, which was reversed by the mixture of GS-Re and lysozyme (Figures 6C–E).

**Figure 7:**
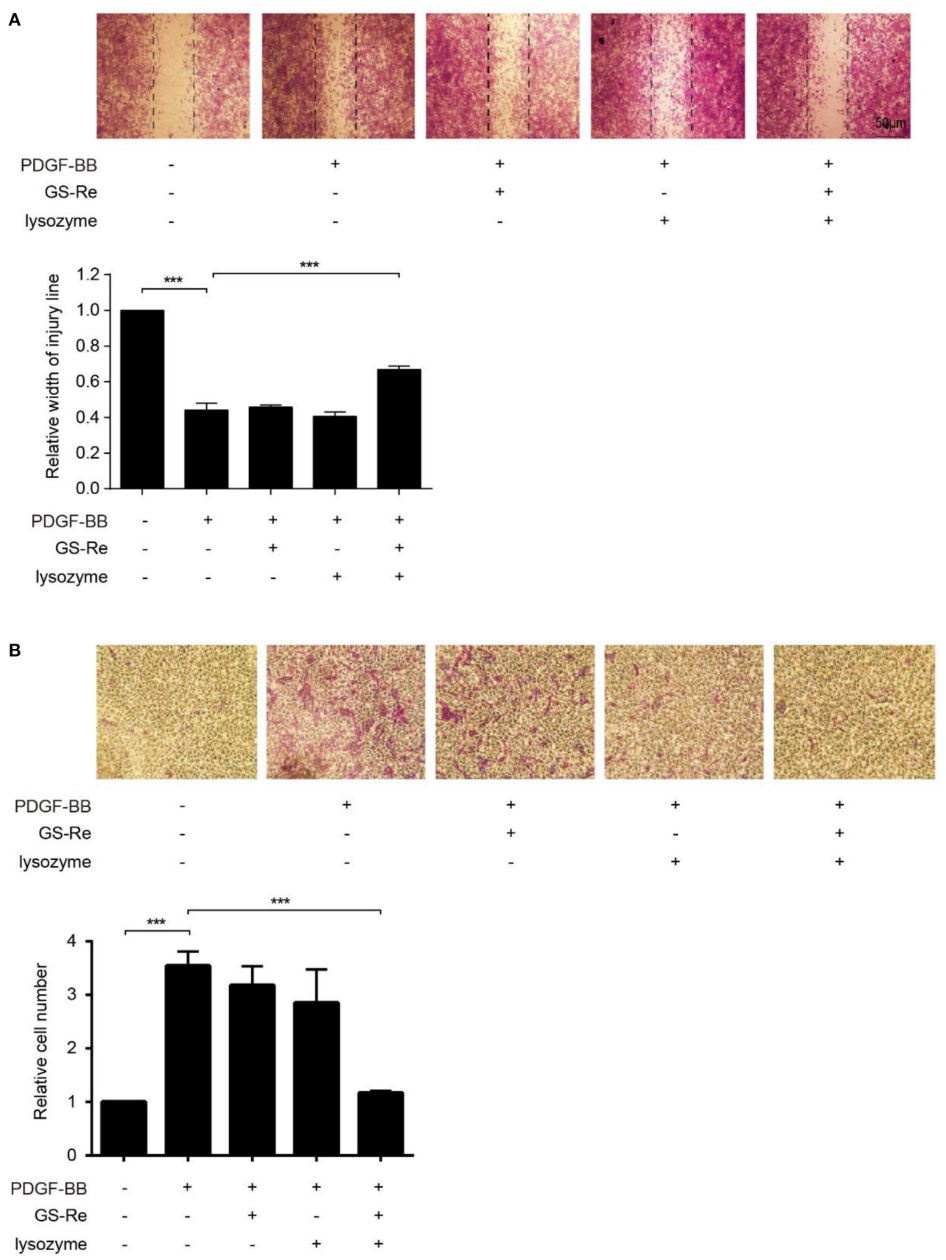
The combined use of GS-Re and lysozyme inhibits VSMC migration. The wound-healing **(A)** and transwell assay **(B)** for the migration of VSMCs, cultured in serum-starved medium containing 12.8 μmol/L GS-Re, 1.067 μmol/L lysozyme, or the mixture of GS-Re and lysozyme (12.8 μmol/L GS-Re+1.067 μmol/L lysozyme) for 6 h, and then stimulated by PDGF-BB or remained untreated. Data are presented as mean ± SD, ***P < 0.001.

Except for proliferation, increased migration is another key process during phenotype transformation of VSMCs (20, 25). To further address the role of PNS and lysozyme synergism in VSMC migration, we performed 2D wound-healing and 3D transwell migration assays. As shown in Figure 7A, the 12:1 mixture of GS-Re and lysozyme inhibited PDGF-BB-induced VSMC wound closure and migration, and GS-Re or lysozyme used alone almost has no effect on it. Results from Boyden chamber transwell assay further confirmed the inhibitory effect of the mixture (Figure 7B). Taken together, these results suggested that lysozyme enhances the activity of GS-Re on the inhibition of VSMC phenotypic transition.

### Mixture of GS-Re and Lysozyme Ameliorates AAA Progression

**Figure 8:**
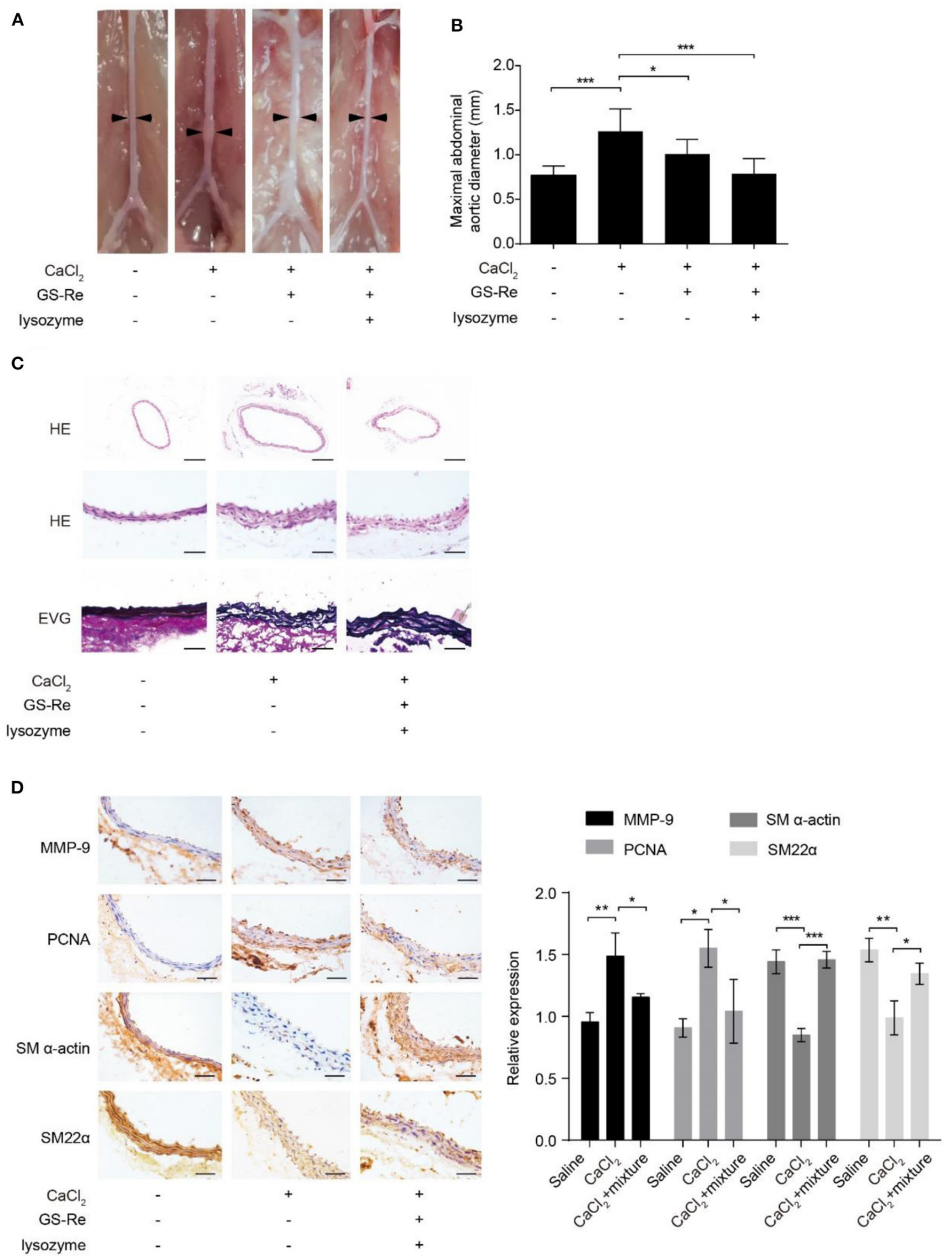
The mixture of GS-Re and lysozyme ameliorates abdominal aortic aneurysm (AAA) progression. **(A)** Representative morphology of aortas in saline or CaCl_2_-induced mice treated by GS-Re or the mixture of GS-Re and lysozyme. **(B)** Quantification of maximal aortic diameters in saline or CaCl_2_-induced mice treated by GS-Re or the mixture of GS-Re and lysozyme. **(C)** Hematoxylin (HE) and Elastic-Van Gieson (EVG) staining of aortas in saline or CaCl_2_-induced mice treated by the mixture of GS-Re and lysozyme. The top bar = 200 μm, the middle and lower bar = 50 μm. **(D)** Immunohistochemistry analysis for the expression of MMP-9, PCNA, SM-α-actin, and SM22α. Bar = 50 μm. Data are presented as mean ± SD, *P < 0.05, **P < 0.01, ***P < 0.001.

The phenotypic transition of VSMCs exhibits at the early onset of the pathology of aortic aneurysms (22). To evaluate the therapeutic potential of the mixture of GS-Re and lysozyme on VSMC phenotypic transition *in vivo*, CaCl_2_-induced C57BL/6J mouse model of AAA was used. Morphometric analysis showed that CaCl_2_ markedly increased the aortic expansion, and compared with the model group, treatment with GS-Re (50 mg/kg/day) alone could slightly reduce the enlargement of arteries, whereas the mixture of GS-Re and lysozyme (50 mg/kg/day, the molar ratio of GS-Re/lysozyme was 12:1) decreased the diameter of arteries significantly (Figures 8A,B). HE and Elastic-Van Gieson (EVG) staining also showed that the mixture of GS-Re and lysozyme preserved vascular tissue integrity and reduced tissue damage and elastin degradation (Figure 8C). Furthermore, the upregulated expression of MMP-9 and PCNA and downregulated expression of SM-α-actin and SM22α were observed *via* immunohistochemical staining in the CaCl_2_-treated group, which were reversed remarkably by the mixture of GS-Re and lysozyme (Figure 8D). These findings indicate that the combination of GS-Re and lysozyme may be involved in the phenotype transformation of VSMCs during the pathogenesis of AAA.

## Discussion

Cardiovascular disease is the leading cause of death worldwide, and compelling evidence has indicated that vascular remodeling represents promising potential therapeutic targets for cardiovascular diseases. As the key cells implicated in vascular remodeling, VSMCs are multifunctional mesenchyme derived from the mesoderm during embryonic development and plays an important role in maintaining vascular tone, blood pressure, and blood flow distribution. Unlike skeletal muscle and cardiomyocytes, VSMCs are highly plastic and susceptible to phenotypic switching from a contractile phenotype to a synthetic, secretory, inflammatory, proteolytic, or even osteochondrogenic phenotype upon various stimulations (1, 2, 4, 26–28), and the dedifferentiated VSMCs are prone to proliferation and migration, produce more inflammatory cytokines and reactive oxygen species, and exhibit greater proteolytic activity than contractile VSMCs (20, 26, 29–32). Therefore, exploring the effective therapeutic strategy for blockade of VSMC phenotype transformation is helpful for treatment of cardiovascular diseases.

Lysozyme, also referred to as muramidase or *N*-acetylmuramic hydrolase, is widely distributed in egg whites of birds and poultry, as well as tears, saliva, plasma, and milk of mammals. In the food or pharmaceutical industries, lysozyme has been widely used against bacteria because of its hydrolysis of β-1,4-glycosidic linkages in specific Gram-positive bacteria wall. Together with the unique characteristics, for instance, higher natural abundance, stability, small size, monomeric protein, positive charge, and drug binding ability, lysozyme has emerged as a model protein for investigating the interaction with different small molecules, pharmaceutical drugs, metal ions, and dyes, as well as in studying the relationship between protein folding and dynamics, structure–function relationship, and cell-to-cell interaction (12, 33). Our previous study has demonstrated that the combination of caffeine and lysozyme through electrostatic interaction increased the concentration of caffeine on the surface of HepG2 cells, showing synergistic effects and accelerating HepG2 apoptosis (18). According to the Traditional Chinese Medicine, PNS improves blood circulation, removes blood stasis, nourishes blood, stops bleeding, eliminates swelling, and relieves pain; so, it has been most widely used to treat cardiovascular and cerebrovascular diseases (6, 34, 35). In the current study, we revealed that 15, 30, and 60 μg/ml PNS did not significantly decrease PDGF-BB-induced viability of VSMCs, and 120 and 240 μg/ml PNS significantly inhibited it. Then, the highest concentration of PNS that did not influence cell viability (60 μg/ml), 60 μg/ml lysozyme, and the mixture of PNS and lysozyme (30 μg/ml PNS + 30 μg/ml lysozyme) were selected to treat VSMCs, and when the VMSCs were treated by the mixture, we found that lysozyme promotes the activity of PNS on the inhibition of VSMC viability. Based on the ability of lysozyme to easily bind with drug molecules and increase the efficacy (17, 18), we predicted that some of the PNS components may play a key role in this process by binding to lysozyme. Results from CCK-8 assay indicated that, among the five main ingredients of PNS, GS-Re might be the only one that reduced VSMC viability through joint application with lysozyme, and GS-Re might play a synergistic role by combing with lysozyme.

Next, to determine the interaction between GS-Re and lysozyme, mass spectrometry was applied. We found that the binding ability of GS-Re and lysozyme increased with the ratio of GS-Re to lysozyme from 4:1 to 12:1, and the concentration ratio of GS-Re to lysozyme was 12:1 as the subsequent experimental condition. We also found that the relative contents of α-helix in lysozyme of supramolecular complex formed by GS-Re and lysozyme were increased, which is not only beneficial for stabilizing the structure of lysozyme and maintaining the exposure of Glu35 and Asp52 active sites to keep the specific biological activity of lysozyme, but also possible to bring the GS-Re–lysozyme complex closer to the surface of the cell membrane to perform synergistic functions. Then, based on the concentration ratio of GS-Re to lysozyme of 12:1, in dose–response studies, we revealed that the mixture of GS-Re and lysozyme did not significantly decrease PDGF-BB-induced viability of VSMCs until they reached 12.8 μmol/L GS-Re+1.067 μmol/L lysozyme, whereas 12.8 μmol/L GS-Re or 1.067 μmol/L lysozyme used along had no effect on VSMC viability.

At the molecular level, we continued to replicate the VSMC phenotype transformation model with PDGF-BB *in vitro*, exhibiting downregulation of differentiated markers (such as SM-α-actin and SM22α) and upregulation of dedifferentiated markers (such as MMP-9 and PCNA), consistent with our and other's previous studies (1, 36–39). The results from qRT-PCR and Western blot demonstrated that the 12:1 mixture of GS-Re and lysozyme (12.8 μmol/L GS-Re+1.067 μmol/L lysozyme) reversed the downregulation of differentiated markers and upregulation of dedifferentiated markers. Our current study also provided evidence that PDGF-BB-induced VSMC migration activity was markedly reduced by the 12:1 mixture *in vitro*.

In addition, many studies have highlighted that VSMC phenotypic transition may be an essential prerequisite for aortic aneurysms formation (22). Therefore, CaCl_2_-induced AAA model was used to test the effect of the mixture of GS-Re and lysozyme. The current study documented that the mixture diminished the development of AAA and presented with the decreased elastin degradation and maintaining the differentiated phenotype of VSMCs in media, implying the obviously synergistic role of GS-Re and lysozyme *in vivo*.

## Conclusion

In summary, the current study identifies the critical role of lysozyme in PNS preventing phenotypic transformation of VSMCs. These findings reveal a mechanism that a small amount of lysozyme significantly promotes the efficacy of PNS by interacting with Gs-Re both *in vitro* and *in vivo*. The finding broadens our understanding of how GS-Re and lysozyme interact with each other, and eventually achieves synergistic effects, which may shed light on the therapeutic strategy for vascular remodeling diseases.

## Data Availability Statement

The raw data supporting the conclusions of this article will be made available by the authors, without undue reservation.

## Ethics Statement

The animal study was reviewed and approved by Hebei Medical University.

## Author Contributions

PL and YZ conducted experiments and wrote the manuscript. YH and LC did Spectral analysis. LC, HY, NC, and HG planned and did *in vitro* experiments, including cell culture, Western blot, cell viability assay, qRT-PCR, and cell migration assay. YH, XG, RW, WS, and YW planned and did *in vivo* experiments, including AAA model, HE, EVG, and IHC staining. All authors contributed to the article and approved the submitted version.

## Funding

This work was supported by the National Key Research Project of China (2019YFC1606400), Major Public Welfare Projects in Henan Province (201300110200), National Key Research Project of Hebei Province (20375502D), Natural Science Foundation of Hebei Province (H2019206212, H2021206427), High-level Talent Funding Project of Hebei Province (A201905006), Fund of National R&D Center for Edible Fungus Processing Technology, Henan University (20200109), the Open Fund from Beijing Advanced Innovation Center for Food Nutrition and Human Health (20182025), and Science and Technology Project of Hebei Education Department (QN2018058).

## Conflict of Interest

The authors declare that the research was conducted in the absence of any commercial or financial relationships that could be construed as a potential conflict of interest.

## Publisher's Note

All claims expressed in this article are solely those of the authors and do not necessarily represent those of their affiliated organizations, or those of the publisher, the editors and the reviewers. Any product that may be evaluated in this article, or claim that may be made by its manufacturer, is not guaranteed or endorsed by the publisher.
